# Orations and Catalogues

**Published:** 1875-10

**Authors:** 


THE following orations received: Address before Medical Association of the State of Alabama, by Dr. J. S. Weatherly. Annual Oration before the Medical and Chirurgical Faculty of Maryland, by J. M. Toner, M.D. Also, Catalogue of the University of Michigan.

